# Azabicyclic vinyl sulfones for residue-specific dual protein labelling†
†Electronic supplementary information (ESI) available: Detailed methods and additional characterisation. See DOI: 10.1039/c9sc00125e


**DOI:** 10.1039/c9sc00125e

**Published:** 2019-03-18

**Authors:** Enrique Gil de Montes, Ester Jiménez-Moreno, Bruno L. Oliveira, Claudio D. Navo, Pedro M. S. D. Cal, Gonzalo Jiménez-Osés, Inmaculada Robina, Antonio J. Moreno-Vargas, Gonçalo J. L. Bernardes

**Affiliations:** a Departamento de Química Orgánica , Facultad de Química , Universidad de Sevilla , C/Prof. García González, 1 , 41012-Sevilla , Spain . Email: ajmoreno@us.es; b Department of Chemistry , University of Cambridge , Lensfield Road , CB2 1EW Cambridge , UK . Email: gb453@cam.ac.uk; c Instituto de Medicina Molecular , Faculdade de Medicina da Universidade de Lisboa , Av. Prof. Egas Moniz , 1649-028 Lisboa , Portugal; d Departamento de Química , Centro de Investigación en Síntesis Química , Universidad de La Rioja , 26006 Logroño , Spain; e CIC bioGUNE , Bizkaia Technology Park, Building 801A , 48170 Derio , Spain

## Abstract

We have developed [2.2.1]azabicyclic vinyl sulfone reagents that simultaneously enable cysteine-selective protein modification and introduce a handle for further bioorthogonal ligation. The reaction is fast and selective for cysteine relative to other amino acids that have nucleophilic side-chains, and the formed products are stable in human plasma and are moderately resistant to retro Diels–Alder degradation reactions. A model biotinylated [2.2.1]azabicyclic vinyl sulfone reagent was shown to efficiently label two cysteine-tagged proteins, ubiquitin and C2Am, under mild conditions (1–5 equiv. of reagent in NaP_i_ pH 7.0, room temperature, 30 min). The resulting thioether-linked conjugates were stable and retained the native activity of the proteins. Finally, the dienophile present in the azabicyclic moiety on a functionalised C2Am protein could be fluorescently labelled through an inverse electron demand Diels–Alder reaction in cells to allow selective apoptosis imaging. The combined advantages of directness, site-specificity and easy preparation mean [2.2.1]azabicyclic vinyl sulfones can be used for residue-specific dual protein labelling/construction strategies with minimal perturbation of native function based simply on the attachment of an [2.2.1]azabicyclic moiety to cysteine.

## Abstract 2

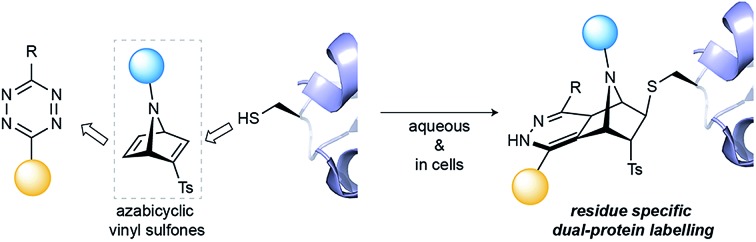
We have developed [2.2.1]azabicyclic vinyl sulfone reagents that simultaneously enable cysteine-selective protein modification and introduce a handle for further bioorthogonal ligation.

## Introduction

For certain biological applications, the installation of two or more distinct synthetic modifications into a protein of interest is desirable.^1^ To introduce two distinct modifications onto a protein two options are possible: (1) site-selectively modify two different amino-acid residues within the protein's sequence^2^,^3^ or (2) use a scaffold that simultaneously displays two different modifications and one handle for site-selective protein modification.^4^,^5^ The latter, which uses multifunctional scaffolds, is perhaps the most straightforward because it does not require orthogonal chemoselective protein reactions.^1^ Senter and co-workers developed a double-drug carrier with a hydrophilic tail that reacts with the protein through a cysteine-reactive handle (maleimide).^5^ In another example, Gonçalves and co-workers reported dichlorotetrazine as a trivalent platform for conjugation to cysteine and showed dual labelling of albumin with a macrocyclic chelator for simultaneous nuclear imaging and a fluorescent probe for imaging.^6^ In addition, Chudasama, Caddick and co-workers have explored dibromopyridazinedione reagents that are able to site-specifically redbridge disulfide bonds and—at the same time—leave two click-reactive handles available for subsequent modifications with fluorophores, PEG derivatives or drugs through strain-promoted or copper-mediated azide–alkyne cycloaddition reactions.^4^,^7^,^8^ Water-soluble allyl sulfones were also shown by Weil and co-workers to rebridge disulfides whilst enabling dual protein functionalisation.^9^ In another example, Swarts and co-workers developed a bicyclo[6.1.0]nonyne-based cyclooctyne reagent for the modification of azide-labelled biomolecules with functional handles that allow both photo-crosslinking and subsequent detection/enrichment of binders.^10^ The latter example is the only one in which the ability to perform dual protein labelling was shown in cells through bioorthogonal labelling, with the others being used to prepare dual-labelled conjugates in the test tube. Thus, the development of simple and robust methodologies that enable the installation of two specific modifications at a single site on a protein specially when one handle can be used for bioorthogonal ligation in cells is an area of prominent interest.

Herein, we present strained [2.2.1]azabicyclic vinyl sulfones as efficient and versatile reagents for residue-specific dual protein labelling. Our design includes a highly reactive and selective alkene handle for bioorthogonal inverse electron demand Diels–Alder (iEDDA) ligation and one easily accessible functionalisation site through *N*-substitution reactions on the azanorbornadiene scaffold.

Vinyl sulfones are useful Michael acceptors and dipolarophiles in cycloaddition reactions for synthesis and medicinal chemistry applications,^11^ and as handles with multi-purpose functions in protein modification.^12^ Their first use for protein modification dates from 1988 in which simple methyl and ethyl vinyl sulfones were used to alkylate thiol, ε-amino or imidazole side-chain amino acids.^13^ Since that time, there have been a number of reports that describe the development of allyl^9^ and vinyl^14^,^15^ sulfones for disulfide rebridging^16^ and cysteine^17^ conjugation methodologies, respectively. Under slightly basic conditions, the sulfhydryl side-chains of the cysteine residues are typically more nucleophilic than the amino groups of lysine and imidazole, and the hydroxyl groups of serine and threonine and thus, selective modification can be achieved.^18^

**Fig. 1:**
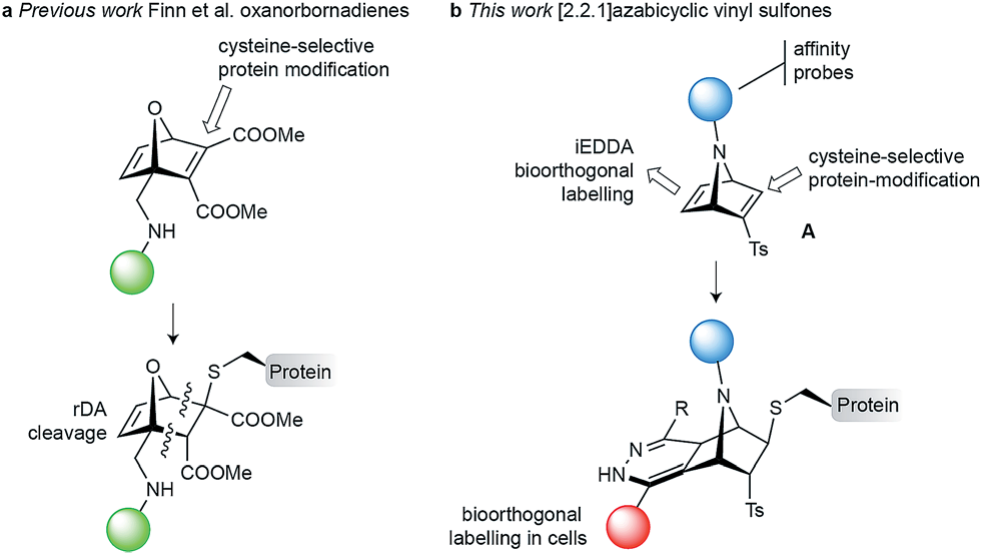
Precedent and proposed linker for cysteine-specific dual protein bioconjugation. (a) 7-Oxanorbornadiene derivatives for cysteine-selective modification and retro Diels–Alder cleavage.^20^–^22^ (b) [2.2.1]Azabicyclic vinyl sulfones highlight the site of cysteine-selective modification, the second reactive handle for bioorthogonal labelling in cells and the explored point-of-attachment.

Strained systems offer an unique opportunity for protein labelling because the significant energy stored in the system is liberated either in part or fully in a click-like bioconjugation process.^19^ Among them, [2.2.1]bicyclic systems, such as norbornenes, exhibit both high reactivity and high stability, and are synthetically accessible. (Hetero)norbornenes and (hetero)norbornadienes can be easily obtained in one step through Diels–Alder reactions between a cyclic diene (cyclopentadiene/furan/pyrrole derivatives) and the appropriate dienophile (electron-deficient alkene or alkyne). Of particular interest are the 7-oxanorbornadiene derivatives developed by Finn and co-workers, which were previously used for site-selective modification of cysteine residues in proteins by conjugate addition (Fig. 1a).^20^,^21^ After cysteine ligation on the protein, oxanorbornadiene undergoes a controllable retro-Diels–Alder (rDA) fragmentation that allowed their use as drug delivery systems.^22^ Strained [2.2.1]bicyclic systems, especially oxanorbornadiene and norbornene systems,^19^ have also been widely used in the field of bioorthogonal chemistry as handles, for example, for fast reactions with tetrazines in iEDDA.^23^ On the contrary, the use of [2.2.1]azabicyclic systems for bioconjugation is rare. A single example showed the use of a 7-azanorbornene as hub molecule for the incorporation of a fluorophore for imaging and biotin as an affinity probe for protein recognition.^24^ This system lacks a handle that would allow for site-selective covalent protein modification.

In this work, we envisioned *N*-substituted azanorbornadienes of type **A**, which combine a vinyl sulfone functionality, an additional reactive double bond and a functionalisable bridging nitrogen as an attractive reagent for cysteine-specific and dual protein labelling (Fig. 1b). The reactive double bond of the resulting azanorbornene upon cysteine modification offers an attractive handle for further bioorthogonal modification at the same residue through iEDDA.^23^,^25^ Finally, the bridging nitrogen can be used to incorporate relevant fragments for protein studies/functions, such as fluorophores, affinity probes or drugs. Thus, with the designed chemical probe we can selectively target cysteine within a protein sequence and functionalise the molecule through simple *N*-derivatisations of the azanorbornadiene and further modify it *in situ* through fast iEDDA reactions with tetrazine reagents.

## Results and discussion

### Development of [2.2.1]bicyclic vinyl sulfones for cysteine modification

**Scheme 1:**
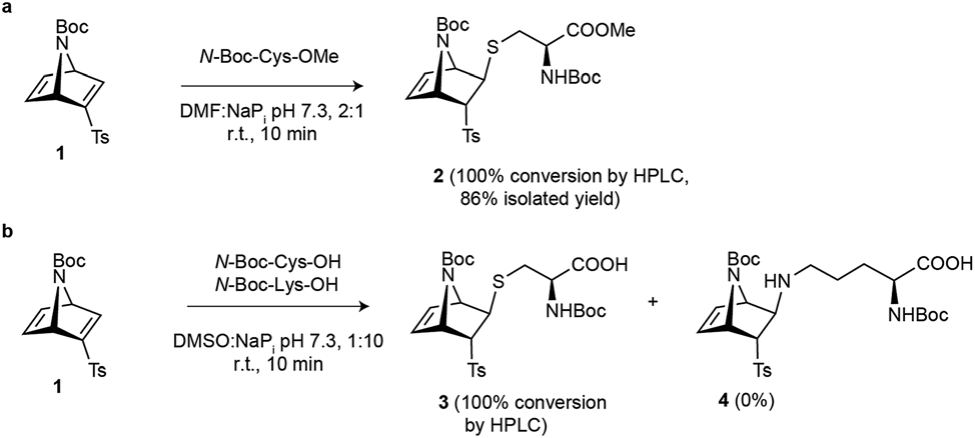
Reaction engineering on amino acid models. (a) Reaction of vinyl sulfone **1** with *N*-Boc-Cys methyl ester derivative. (b) Competition experiment that shows the chemoselective nature of the reaction between [2.2.1]azabicyclic vinyl sulfone **1** and cysteine.

Based on a reported procedure,^26^,^27^ we first carried out the synthesis of racemic azanorbornadiene **1** through a Diels–Alder reaction between inexpensive *N-tert*-butyloxycarbonyl (Boc)-pyrrole and ethynyl *p*-tolyl sulfone. We then tested the reactivity of azanorbornadiene **1** towards cysteine by using *N*-Boc-Cys-OMe as a model system. When a stoichiometric amount of bicyclic system **1** was reacted with the cysteine model in a buffered mixture of NaP_i_ (100 mM, pH 7.3) and *N*,*N*-dimethylformamide (DMF) at room temperature for 10 min, complete conversion to the corresponding *trans* thioether adduct was detected (Scheme 1a). This experimental data confirmed the high reactivity of this particular vinyl sulfone embedded in the strained 7-azanorbornadiene skeleton towards thiol-Michael addition. The *exo*-facial preference of the nucleophilic attack is well documented in other [2.2.1]azabicyclic systems.^27^–^30^ Furthermore, to attest the chemoselectivity of our strained [2.2.1]azabicyclic reagent we performed a competition experiment between *N*-Boc-Cys and *N*-Boc-Lys. Notably, under our experimental conditions [buffered mixture of NaP_i_ (50 mM), pH 7.3, dimethylsulfoxide (DMSO), room temperature, 10 min] only the Cys adduct was obtained and no modification of the ε-amino group of lysine was detected (Scheme 1b and Fig. S6†). Together our data show that [2.2.1]azabicyclic vinyl sulfones can react rapidly under aqueous conditions in a chemo- and stereoselective manner with cysteine residues.

### Stability of cysteine-[2.2.1]azabicyclic vinyl sulfone towards rDA

Having shown the chemoselectivity of **1** towards cysteine residues, we then studied the stability of the formed thioether because we were concerned by some rDA reports of cleavage with similar bicyclic systems.^22^,^31^–^34^ Thus, fragmentation of azanorbornene **2** into *N*-Boc-pyrrole and thio-vinyl sulfone **9** was studied by ^1^H NMR spectroscopy at 37 °C in CDCl_3_ (Table 1, entry 1; see Fig. S8 and S9† for NMR spectroscopic monitoring). Compound **2** has a half-life of ≈16 h, which is nearly half of the longest half-life reported for the corresponding oxanorbornadiene derivative.^22^ As reported, the rDA fragmentation has a tuneable rate depending upon the substituents of the norbornene so we decided to modulate the stability of our reagent. To prove the concept, we introduced two bridgehead methyl groups on the bicyclic system (vinyl sulfone **5**, ESI† for synthetic details), which significantly decreased the half-life (<5 min) of the corresponding cysteine adduct (Table 1, entry 2). In fact, in this case, the corresponding bicyclic adduct was not observed and afforded directly the rDA products. Next, instead of modifying the diene structure of the pyrrole ring, we decided to change the protection of the amino group from Boc in **1** to an acetyl group in **7** (ESI† for synthetic details), which doubled the half-life of the cysteine adduct **8** (37 h; Table 1, entry 3). The stabilising effect was even more predominant when we changed to more polar DMSO-*d*_6_, which resulted in a half-life of ≈59 h, which is nearly 4 times higher than the initial conditions (Table 1, entry 4). Moreover, when a mixture of DMF and buffered aqueous solution was chosen to test the potential rDA, after 59 h at 37 °C only 37% of adduct **8** had decomposed into the rDA products.

**Table 1:** rDA studies of azabicycle-cysteine adducts

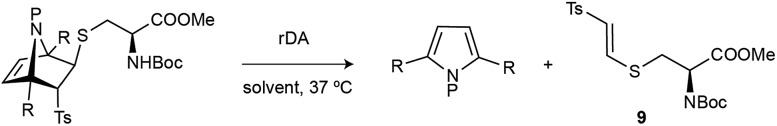						
Entry	Vinyl sulfone^*a*^	Azabicycle-Cys adduct	**R**	**P**	Solvent	Half-life
1	**1**	**2**	H	Boc	CDCl_3_	16.4 h
2	**5**	**6** ^*b*^	Me	Boc	CDCl_3_	<5 min
3	**7**	**8**	H	Ac	CDCl_3_	37 h^*c*^
4	**7**	**8**	H	Ac	DMSO-*d*_6_	59 h

^*a*^See ESI for details on the synthesis of vinyl sulfones **5** and **7**.

^*b*^Azabicycle-cysteine adduct **6** was not observed because it decomposes directly to give **9** and *N*-Boc-2,5-dimethylpyrrole.

^*c*^ *t*_1/2_ = 40 h; CDCl_3_ is pre-neutralized with K_2_CO_3_.

Quantum mechanical calculations on model compounds (Fig. S12 in the ESI†) reproduced the experimentally observed slight slowing down of the reaction when replacing the Boc by the Ac group, and when using a more polar solvent (DMSO *vs.* CHCl_3_), reflecting the high nonpolar character of the rDA transition structures. However, and unlike in the case of Finn's oxanorbornadiene thiol and amine adducts, which have different substitution patterns and exhibit much more asynchronous transition structures as studied by Houk,^35^ the difference in reactivity of the bridgehead-methylated and non-methylated analogues was not so apparent computationally, and only using nearly complete models could reproduce the experimental trend. While the rDA can take place under physiologically relevant conditions, our data shows that this reaction is very slow (>59 h) for conjugates formed through modification of cysteine with [2.2.1]azabicyclic vinyl sulfone reagents, which is promising for their potential utility to build conjugates for *in vivo* studies.

### [2.2.1]azabicyclic vinyl sulfones for residue-specific protein labelling

**Fig. 2:**
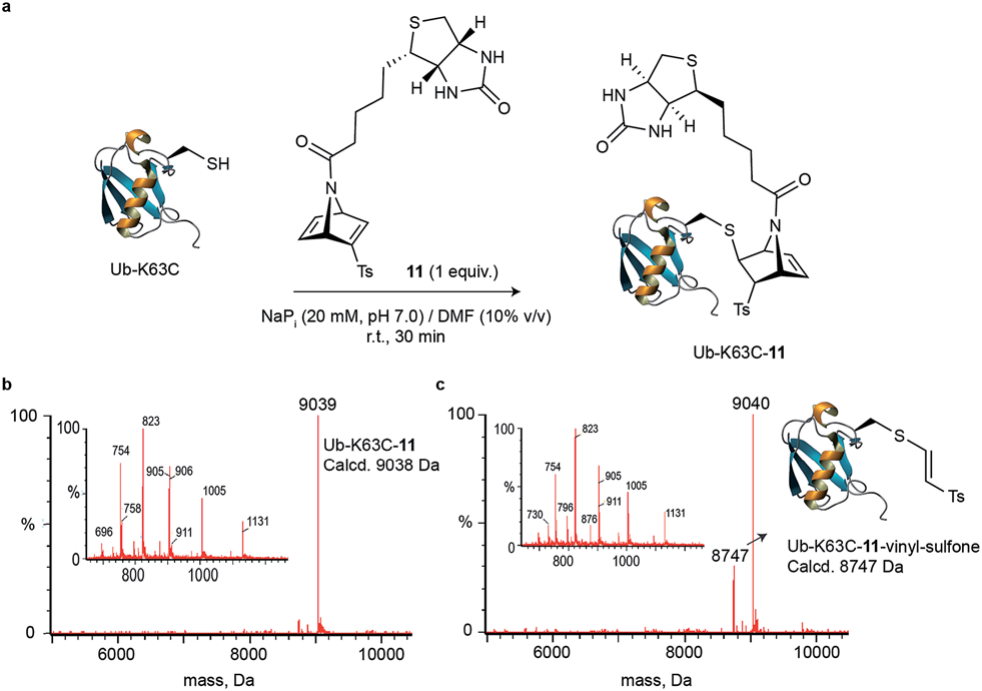
Cysteine site-selective bioconjugation with [2.2.1]azabicyclic reagent **11**. (a) Reaction between Ub-K63C and **11**. General reaction conditions: Ub-K63C reacted with **11** (1 equiv.) in NaP_i_ (20 mM, pH 7.0) at 25 °C for 30 min. (b) Deconvoluted mass spectrum obtained from the represented reaction. (c) Deconvoluted mass spectrum obtained after incubation of Ub-K63C-**11** in plasma for 24 h at 37 °C.

We decided to evaluate whether our findings on small molecules could be translated into cysteine-tagged proteins. We started by adding d-biotin to the bridging nitrogen of the strained [2.2.1]azabicyclic vinyl sulfone scaffold for potential use in imaging or enrichment experiments. The bridging nitrogen is ideal as a point of attachment because it is not expected to influence the reactivity of the vinyl sulfone towards thiol-Michael addition. Briefly, acidic Boc-deprotection of **1** followed by acylation with d-biotin acid chloride **10** afforded biotinylated azanorbornadiene **11** in 75% overall yield (see Scheme S1 in the ESI†). An identical synthetic route may be used to incorporate other motifs routinely used for protein modification including PEG, drugs or fluorophores (*e.g.* dansyl moiety, see Scheme S2,† compound **12**). As a model protein for labelling, we decided to produce a mutant of a multivalent protein–ubiquitin (Ub-K63C) – that has been engineered to feature a surface-exposed cysteine residue at position 63 (for further details see the ESI†).^36^ By using an equimolar amount of biotin-functionalized [2.2.1]azabicyclic vinyl sulfone **11** in NaP_i_ buffer (20 mM, pH 7.0) and DMF as co-solvent (10%) at room temperature for 30 min, Ub-K63C was fully converted into a single and homogenous product (Fig. 2a and b) as analysed by LC-MS. The crude reaction was then subjected to excess Ellman's reagent and after 1 h at 37 °C, no change was noted, which confirms that all cysteine was consumed during the Michael reaction with vinyl sulfone **11** (see Fig. S18†), and further confirmed the chemoselectivity of our method. Importantly, we showed that the Ub-K63C-biotin conjugate is relatively stable in plasma media. After 24 hours incubation at 37 °C with plasma, only modest degradation of the product (<20%) was observed as a result of retro-Diels–Alder reaction (see Fig. S19†). Moreover, the original thioether linkage remained unaltered and did not exchange with other biological thiols present in plasma media. Our data show a cysteine-specific protein modification with a [2.2.1]azabicyclic vinyl sulfone that bears a synthetic modification inserted at the bridging nitrogen of the reagent that leaves the remaining alkene unreacted, which may be further used for iEDDA ligation in a test tube or in cells.

### Imaging C2Am-**11**-labelled apoptotic cells

**Fig. 3:**
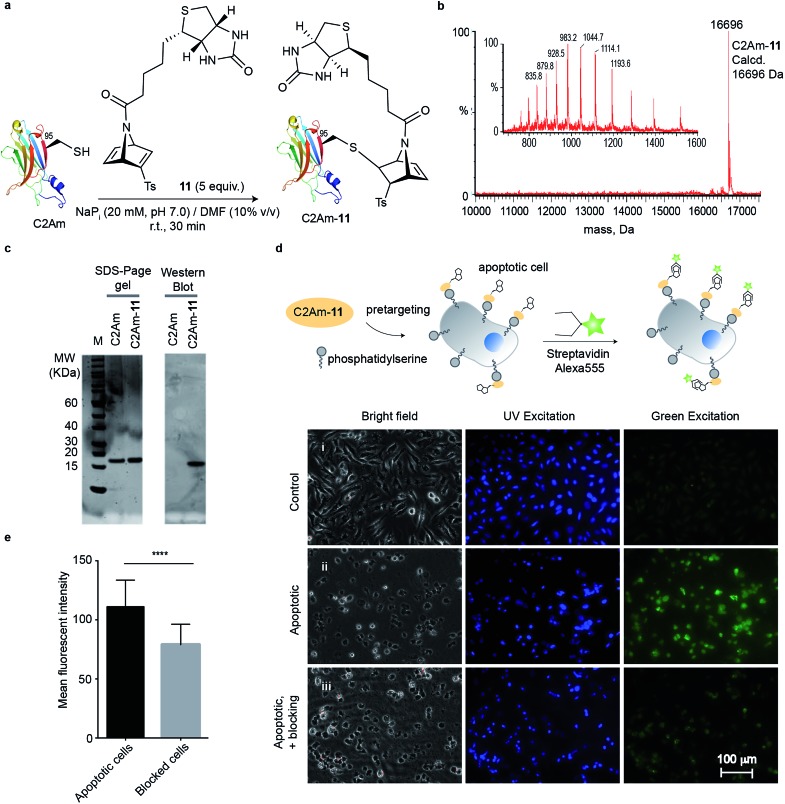
Imaging of apoptotic HeLa cells with C2Am-**11**. (a) Reaction between C2Am and **11** in NaP_i_ (20 mM, pH 7.0) at 25 °C for 30 min. (b) Deconvoluted mass spectrum obtained from a representative reaction. (c) Treatment of C2Am-**11** with Streptavidin-Alexa555 after SDS-PAGE and membrane transfer gave a single fluorescent band as detected by Western blot that confirms biotinylation of C2Am and binding of the fluorescent streptavidin to the biotin. Lanes 1 and 2, Spyro Protein Ruby staining (SDS-PAGE); lanes 3 and 4, fluorescence at excitation and emission wavelengths for Alexa 555 (Western Blot membrane). (d) Epifluorescent images of non-apoptotic (control) and apoptotic HeLa cells after labelling with C2Am-**11** + Streptavidin Alexa555. Blocking studies were performed by pre-incubating apoptotic cells with an excess of non-fluorescent C2Am before incubation with C2Am-**11** + Streptavidin Alexa555. Apoptotic cells are shown in green. Blue represents Hoechst-stained nuclei. (e) MFI from apoptotic and blocked cells determined with ImageJ (average of 30 regions of interest). Statistical significance was determined by paired *t* test by using GraphPad Prism (****, *P* < 0.0001).

Encouraged by our results, we envisioned to apply our biotin-functionalised reagent for cysteine-selective protein modification and further labelling with a streptavidin probe for imaging. For these studies we used the C2A domain of Synaptotagmin-I (C2Am) that binds to phosphatidylserine (PS), which is localized on the external leaflet of the plasma membrane during apoptosis. In previous studies a C2Am mutant that has a single cysteine at the position 95 for modification with a radionuclide, a fluorescent probe, or a magnetic resonance-detectable tag was used for imaging apoptotic tissues *in vivo*.^37^ Thus, we reacted this protein mutant with compound **11** by using similar conditions to those described above (5 equiv., 20 mM of NaP_i_ buffer/10% DMF at pH 7.0, room temperature, 30 min reaction) to give a single homogenous product (Fig. 3a and b) determined by LC-MS. The efficiency of the biotinylation reaction was also examined by Western blot. Non-modified C2Am (control) and biotinylated protein C2Am-**11** were resolved by SDS-PAGE. Biotinylation was confirmed by using a streptavidin fluorescent probe (Streptavidin-Alexa555) after transfer to a polyvinylidene difluoride membrane (Fig. 3c). We then tested if this C2Am derivative retains its inherent functionality of binding to the PS phospholipid on the surface of apoptotic cells. Therefore, C2Am-**11** was used for imaging apoptotic cells by pre-targeting followed by labelling with Streptavidin-Alexa555.^38^ Apoptosis in HeLa cells was induced by treatment with actinomycin D (Act D, 1 μM, 12 h). Following treatment with Act D, cells were washed and incubated with C2Am-**11** (6 μM, 20 min) at 37 °C. After pre-incubation, apoptotic cells were further washed and incubated with the counterpart of biotin, Streptavidin-Alexa555 (2.5 μL of 1 mg mL^–1^, 20 min). Blocking studies were performed by incubating apoptotic cells with a large excess of non-modified C2Am and then with biotinylated surrogate C2Am-**11** and Streptavidin-Alexa555. Fluorescent microscopy images showed staining of only apoptotic cells by C2Am-**11** + Strep-Alexa555, whereas healthy control cells remained unstained (Fig. 3d). Moreover, in the blocking experiments, in which cells were first incubated with the non-labelled C2Am, a significant decrease of the mean fluorescence intensities (MFI) was observed (Fig. 3e). These results attest the ability of modified C2Am-**11** to bind to PS to enable specific labelling of apoptotic cells.

### Reactivity of cysteine-[2.2.1]azabicyclic reagents towards tetrazines

After having studied the reactivity, stability and utility of the cysteine attachment point of [2.2.1]azabicyclic vinyl sulfones, we explored the possibility of a second reactive handle for site-specific modification with the addition of a proper counterpart. In our case, the subsequent modification would take advantage of the remaining double bond present in the bicyclic system by its reaction with tetrazines through iEDDA.

**Fig. 4:**
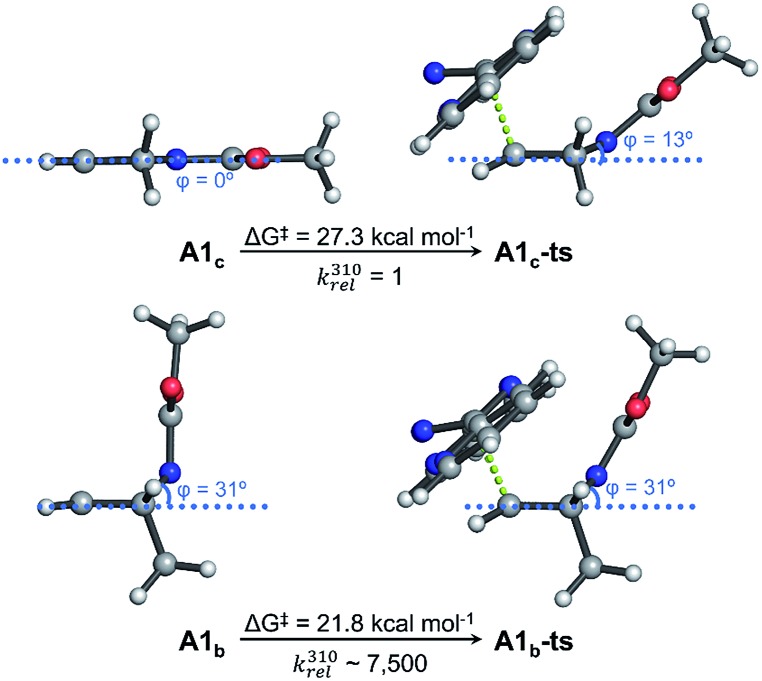
Kinetic studies for the iEDDA reaction between azanorbornene **2** and tetrazines **13a–13c**. Lowest-energy conformations calculated for *N*-Moc-protected (Moc = methyl carbamate) cyclic 2,5-dihydro-1*H*-pyrrole (**A1_c_**) and bicyclic azanorbornene (**A1_b_**) models and their iEDDA reactions with tetrazine **13a** calculated with PCM_DMF_/M06-2X/6-31+G(d,p). Five-membered ring puckering is represented by the dihedral angle *φ* and relative reaction rate constants at 37 °C (310 K, *k*310rel) are calculated from activation free energies (Δ*G*^‡^). A significant distortion of the dihydropyrrole ring is required to achieve the transition state geometry in **A1_c_**, while in **A1_b_** such distortion is already developed up in the dienophile, translating into a reaction rate acceleration of several orders of magnitude.

Based on a precedent with reactions between azanorbornene derivatives and tetrazines,^24^ we evaluated the reactivity of thioether bicyclic system **2** with different tetrazines (**13a–13c**) by monitoring the decrease of tetrazine absorbance over time.^23^ We found that the dienophile present in **2** can quickly and easily undergoes iEDDA reactions with tetrazines.^23^ Considering the properties of the diene to perform an iEDDA most electron-deficient tetrazines **13a** and **13b** resulted in a faster reaction, whereas more electron-rich tetrazine **13c** reacted an order of magnitude slower (Table 2). As demonstrated by quantum mechanical calculations (Fig. 4 and S13†), and contrary to what is commonly assumed, the superior reactivity of *N*-substituted azanorbornenes compared to their non-bicyclic analogues *does not arise from any special strain at the double bond*, as it is the case for other highly strained π-systems such as *trans*-cyclooctene or cyclooctyne. This can be deduced from the very similar double bond lengths and angles in non-bicyclic and bicyclic analogues. Moreover, such apparent activation of the alkene group strongly depends on the type of substitution at the bridging atom. While *N*-alkyl azanorbornenes or norbornenes show nearly the same (or even slower) calculated iEDDA reaction rates with tetrazine **13a** than their non-bicyclic five-membered analogues (1-alkyl-2,5-dihydro-1*H*-pyrroles and cyclopentene, respectively), calculated activation barriers for *N*-carbonyl (carbamate or amide)-substituted azanorbornenes are 5–6 kcal mol^–1^ smaller than those of their non-bicyclic counterparts, resulting in a 3–4 orders of magnitude acceleration. The source of this acceleration is the distortion of the otherwise planar 1-carbonyl-2,5-dihydro-1*H*-pyrrole ring into a puckered conformation closely resembling that at the transition state, similarly to what described for norbornenes *vs.* (*Z*)-but-2-ene or cyclohexene.^39^Fig. 4 shows that the iEDDA reaction causes a puckering of the planar five-membered 2,5-dihydro-1*H*-pyrrole ring in model system **A1_c_** of ≈13° at the transition state, while for bicyclic analogue **A1_b_** the structural changes at the transition state are minimal. This reflects the pre-distorted nature of azanorbornenes and thus their superior ability to achieve transition state geometries through lower activation barriers, which ultimately translates into reaction rate acceleration.

**Table 2:** Kinetic studies for the iEDDA reaction between azanorbornene **2** and tetrazines **13a–13c**

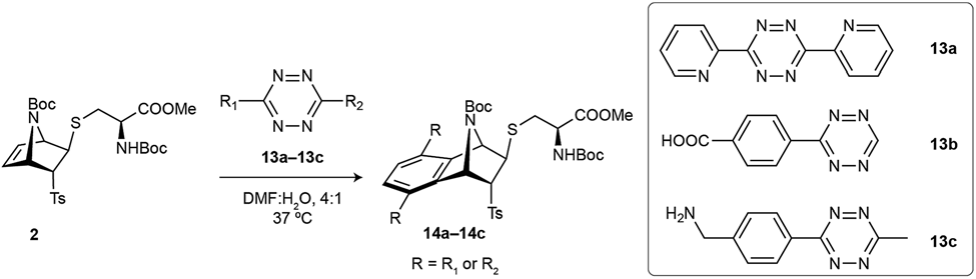		
Entry	Tetrazine	*k* × 10^–2^ M^–1^·s^–1^
1	**13a**	2.6 ± 0.1
2	**13b**	3.1 ± 0.1
3	**13c**	0.143 ± 0.005

These data highlight the suitability of strained [2.2.1]azabicyclic vinyl sulfone reagents to site-specifically modify cysteine residues whilst simultaneously leaving a reactive alkene free for further iEDDA labelling.

### [2.2.1]azabicyclic vinyl sulfones for residue-specific dual protein labelling

With the chemoselectivity towards cysteine residues evaluated (one attachment point studied with an affinity tag, and the viability of chemically modifying the second reactive handle in our [2.2.1]azabicyclic vinyl sulfones with tetrazines appraised), we then needed to see whether this last achievement was also reproducible in a physiological environment. This would allow us to demonstrate the dual-labelling character present in this vinyl sulfone.

**Fig. 5:**
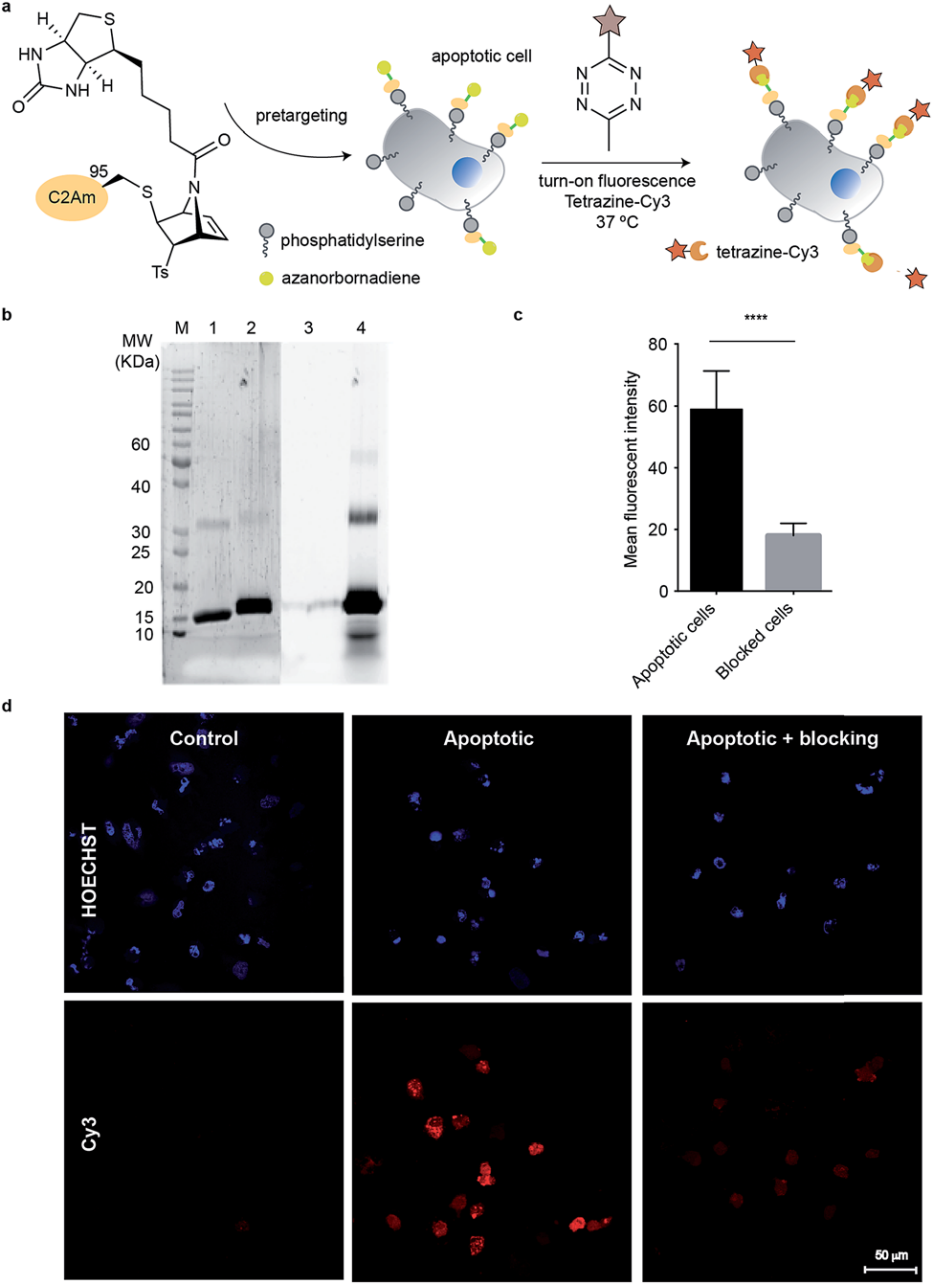
Imaging apoptotic cells with tetrazine-Cy3 after pre-targeting with bioconjugate C2Am-**11**. (a) Scheme of the general procedure for imaging cells. (b) Fluorescent images of non-apoptotic (control) and apoptotic HeLa cells after labelling with C2Am-**11** + Tz-Cy3. Blocking studies were performed by pre-incubating of apoptotic cells with an excess of non-fluorescent C2Am before incubation with C2Am-**11**. (c) MFI from apoptotic and blocked cells determined with ImageJ (average of 30 regions of interest). Statistical significance was determined by paired *t* test by using GraphPad Prism (****, *P* < 0.0001). (d) Treatment of C2Am-**11** with 6-methyl-tetrazine-sulfo-Cy3 gave a new fluorescent band as detected by SDS-PAGE (lanes 2 and 4) that is consistent with the fluorogenic tetrazine bonding to the azanorbornadiene. Control lanes 1 and 3 refer to incubation of unmodified C2Am with Tz-Cy3 under the same conditions. Lanes 1 and 2, Spyro Protein Ruby staining; lanes 3 and 4 fluorescence from excitation/emission of Cy3.

We planned to pre-target apoptotic cells with our previously synthesized bioconjugate with the affinity tag (C2Am-**11**) and then use fluorogenic and commercially available 6-methyl-tetrazine-sulfo-Cy3 (Tz-Cy3) to functionalise the remaining double bond (Fig. 5a). The protocol for targeting cells with C2Am-**11** was similar to the one mentioned above. After incubation with the bioconjugate, cells were washed and further incubated with fluorogenic tetrazine for 90 min. Tetrazine labelling of pre-targeted C2Am-**11** allowed ready visualization of cells rendered apoptotic by treatment with Act D (Fig. 5a). Similarly, cells previously blocked with “native” C2Am, and then labelled with C2Am-**11** + Tz-Cy3 following the same protocol, showed a significant decrease of fluorescence (Fig. 5c). These experiments confirm that the specific affinity of C2Am-**11** for apoptotic cells is retained and the double bound is accessible by a tetrazine fluorophore in a cellular environment (Fig. 5d). Reactivity of the double bond towards the fluorescent tetrazine was studied by SDS-PAGE (Fig. 5b). After 90 min a fluorescent band that corresponded to C2Am-**11**-Tz-Cy3 modified protein was observed (Fig. 5c and d). We further tested the versatility of this system for dual modification of proteins. Reaction with precursor **12** (dansylglycine-azanorbornadiene) enabled installation of a fluorescent tag on ubiquitin that could be further modified by reaction with a tetrazine compound (accessed by MS and SDS-PAGE in-gel fluorescence; Fig. S20 and S21†). Overall, the azanorbornadiene handle can be used as a platform for double modification of the same protein without changing its native properties.

## Conclusions

In this work, we explored a [2.2.1]azabicyclic vinyl sulfone for cysteine-specific dual protein labelling. We have demonstrated the attachment of synthetic modifications through *N*-substitutions of a pyrrole ring and showed the utility of having a second reactive handle to perform bioorthogonal ligation in cells through a site-specific reaction between a bicyclic alkene and a tetrazine. Furthermore, we have proven the stability of the anchorage in plasma, and demonstrated that the modified proteins retain their initial function. The simplicity and synthetic accessibility of these azanorbornadienes makes them ideal reagents for cysteine-specific dual protein modification. This strategy may be used for example for the attachment of two different drugs to a targeting protein/antibody, the incorporation of different tags for target identification or even a cytotoxic molecule and a fluorophore for following cancer treatment.

## Conflicts of interest

There are no conflicts to declare.
